# The Potential Impact of Adjunct Digital Tools and Technology to Help Distressed and Suicidal Men: An Integrative Review

**DOI:** 10.3389/fpsyg.2021.796371

**Published:** 2022-01-04

**Authors:** Luke Balcombe, Diego De Leo

**Affiliations:** Australian Institute for Suicide Research and Prevention, School of Applied Psychology, Griffith University, Brisbane, QLD, Australia

**Keywords:** high-risk and vulnerable men, suicide prevention, digital mental health interventions, digital tools and technology, self-help, human-computer interaction, technology-enabled care, integrated digital platform

## Abstract

Suicidal men feel the need to be self-reliant and that they cannot find another way out of relationship or socioeconomic issues. Suicide prevention is of crucial importance worldwide. The much higher rate of suicide in men engenders action. The prelude is a subjective experience that can be very isolating and severely distressing. Men may not realize a change in their thinking and behaviors, which makes it more difficult to seek and get help, thereby interrupting a “downward spiral”. Stoicism often prevents men from admitting to their personal struggle. The lack of “quality” connections and “non-tailored” therapies has led to a high number of men “walking out” on traditional clinical approaches. But there are complicated relationships in motivations and formative behaviors of suicide with regards to emotional state, psychiatric disorders, interpersonal life events and suicidal behavior method selection. Middle-aged and older men have alternated as the most at-risk of suicide. There is no one solution that applies to all men, but digital tools may be of assistance (e.g., video conferences, social networks, telephone calls, and emails). Digital interventions require higher levels of effectiveness for distress and suicidality but self-guided approaches may be the most suitable for men especially where linked with an integrated online suicide prevention platform (e.g., quick response with online chats, phone calls, and emails). Furthermore, technology-enabled models of care offer promise to advance appropriate linking to mental health services through better and faster understanding of the specific needs of individuals (e.g., socio-cultural) and the type and level of suicidality experienced. Long-term evidence for suicidality and its evaluation may benefit from progressing human computer-interaction and providing impetus for an eminent integrated digital platform.

## Background

Suicide prevention is a primary global health concern. After a pattern of steady rise since the mid-2000s, suicide rates did not generally increase in the first months of the COVID-19 pandemic (Sinyor et al., 2021). However, more needs to be known about vulnerable populations and the effects of long-term economic and mental health stress (Sinyor et al., 2021). Prior to the current pandemic, men were at a significant high-risk of suicide across all ages worldwide (1.5–10 times more prevalent than women) which called for the development of reliable knowledge for suicide preventive strategies to diagnose depressed and suicidal men (Sher, 2015; World Health Organization [WHO], 2021) as well as a better appreciation of this immense public health challenge (Sher, 2019). Pandemic-related stress (e.g., disease risk, ongoing economic uncertainty, loneliness, and lifestyle changes) have exacerbated men’s high-risk for depression and suicidal ideation (Ellison et al., 2021). Lower prevalence rates of common mental health problems (i.e., anxiety and depression) in men should not be inferred that there is less overall experience of such (Gough et al., 2021). Instead, it was suggested that reporting of psychological symptoms is limited by hegemonic masculinities related to self-reliance and not expressing emotions. The preliminary validation of the Gender-Sensitive Depression Screening (Möller-Leimkühler and Mühleck, 2020) led to the proposal that the identification of atypical symptoms of depression may be useful for suicide prevention evaluations in forensic practice (Streb et al., 2021). Psychological screening analyses found non-reporting of affective symptoms as well as external behaviors (e.g., drinking alcohol or being aggressive to fit in with masculine norms and for masking or coping purposes) (Streb et al., 2021). In-roads are being made toward emotional intelligence in men. In-person activity-based initiatives (e.g., Men’s Sheds) have brought together mainly older men to support each other whilst mental health support online offers promise in targeting communities of men (e.g., lifestyle and ethnic groups) (Gough et al., 2021).

Men increased their seeking of mental health care (at a higher rate than women for family and relationships) during COVID-19 (i.e., there was a 79% increase in virtual mental health care visits between January and September 2020) (Ellison et al., 2021). The current pandemic has contributed to the increased interest in and use of phones, computers, and other electronic devices for telehealth (Barnett et al., 2021) as well as technologies for the identification and treatment of mental ill-health across varied populations (Torous et al., 2020; Sorkin et al., 2021). Evidence of effectiveness for digital interventions was generally established for common disorders (i.e., anxiety and depression, particularly for adolescents and young people) (Lehtimaki et al., 2021). There is capacity for digital mental health to assist in serving the marginalized and underserved (Schueller et al., 2019) and effectively fill unmet needs for mental health care (Wind et al., 2020; The Lancet Digital Health, 2021). However, there are challenges with ethics, security, equity, user retention, and evaluation (Balcombe and De Leo, 2021a). Human support via paraprofessionals may increase the reliability and scalability of digital mental health interventions and help in overcoming user uptake, engagement, and dropout issues (especially in routine clinical settings) (Rosenberg et al., 2021).

A review of gender differences in the effectiveness of suicide prevention efforts found very few cases, none of which described digital mental health, and most were maximally beneficial to females (Hamilton and Klimes-Dougan, 2015). A systematic review and meta-analysis of randomized controlled trials (RCTs) found self-guided digital interventions are effective for directly targeting individuals’ suicidal ideation (immediately after post-intervention) (Torok et al., 2020). An emerging indication of a late debut and ambiguous presentation of suicide-related communication in boys led to the suggestion for future research to explore online suicide-related communication and the potential for suicide prevention on digital platforms (Balt et al., 2021). To our knowledge, there is no literature specifically focusing on gender differences in the efficacy of digital assistance in suicide prevention. Therefore, theoretical and empirical samples of the literature were explored with the aim of appraising and synthesizing the potential impact of digital mental health tools and technologies to assist in the provision of faster and better service to distressed and suicidal men.

## Methods

This integrative review purposively sampled from Sage, ScienceDirect, Google Scholar, and CrossRef databases, reference lists of relevant reviews as well as three mental health websites and the WHO website. Search terms included: men, mental health, suicide prevention, digital mental health, digital tools, technology, digital interventions, digital phenotyping, telehealth, phone, email, internet, virtual reality, gender differences, human-computer interaction, and combinations of these terms. The authors independently assessed all abstracts against the inclusion and exclusion criteria according to the 5-step amendment (Balcombe and De Leo, 2021b; see Table 1) of a modified integrative review framework (Whittemore and Knafl, 2005). This methodology was applied to critically evaluate and synthesize the reported outcomes of empirical and theoretical literature converging “suicide in men” and “digital mental health” (i.e., with a focus on effectiveness, feasibility, accessibility, socio-cultural inclusion, rigor and readiness for adoption and upkeep in suicide prevention). The next section presents a summary of results followed by key concepts to provide clear and concise synthesis of the body of knowledge and recommendations.

**TABLE 1:** Five step integrative review literature search method.

(1) Problem identification
(2) Literature search
• Participant characteristics
• Reported outcomes
• Empirical or theoretical approach
(3) Author views
• Clinical effectiveness
• User impact (feasibility/accessibility)
• Social and cultural impact
• Readiness for clinic or digital solutions adoption
• Critical appraisal and evaluation
(4) Determine rigor and contribution to data analysis
(5) Synthesis of important foundations or conclusions into an integrated summation

## Summary of Men’s Mental Health and Suicidality

Scientific research has yet to establish the underlying causes of suicide in men. More needs to be known about the complex relationship between mental ill-health, its comorbidities and the biopsychosocial factors that influence suicidality. Preliminary findings from Australian qualitative research with 35 suicide survivors described risk factors (i.e., disrupted mood, unhelpful stoic beliefs and values, avoidant coping strategies, and stressors) that led to suicidal behaviors; misinterpretation of changes in their thinking and behavior disabled identification of opportunities to interrupt suicide progression (a downward spiral) (Player et al., 2015). Furthermore, there was a lack of distraction, practical and emotional supports as well as the need for effective and tailored professional intervention (Player et al., 2015). Other Australian researchers have contributed quantitative evidence with their suggestions about male vulnerability and suicidal ideation: self-reliance stood out as a risk factor (Pirkis et al., 2016) in addition to family conflict, break-up of a relationship, difficulty finding a job, legal troubles, major loss of property and serious personal injury (Currier et al., 2016). An American perspective agreed on some aspects of suicidal behavior – the impact of socioeconomic issues and divorce but added parental alienation and pathophysiology issues – testosterone) (Sher, 2018).

There is a need to better understand and measure the motivations of those who die by suicide. A study that applied the Inventory of Motivations for Suicide Attempts established reliability for a two-factor structure – firstly for intrapersonal motivations related to ending emotional pain and secondly for interpersonal motivations related to communication or help-seeking (May and Klonsky, 2013). The former was associated with greater intent to die, while the latter was associated with less lethal intent and greater likelihood of rescue. Clinical relevance was proposed in a four-factor analytic study with a patient sample exhibiting high suicidality (Hayashi et al., 2017). Intrapersonal and interpersonal directedness classifications were used in support of the hypothesis that motivation for suicidal behavior is central to its development (Hayashi et al., 2017). A model provided understanding of the formative processes of suicidal behavior. Future studies were suggested to investigate the complicated relationships with emotional state, psychiatric disorders, interpersonal life events and suicidal behavior method selection.

Men have varied perceptions, expectations, attitudes, and behaviors with regards to mental health. Norms of masculinity associated with depression in the Australian cultural context focused on independence, invulnerability, avoidance of negative emotions, and stoicism (Rice et al., 2011) – the latter personality trait is also associated with suicide (Alston, 2012; Witte et al., 2012). The most at-risk for suicide age groups change over time (and vary between countries). In the previous decade, older men were reported to predominate suicide deaths in a global context – only clues were provided as to their vulnerability (e.g., rigid and traditional masculine sense of self and coping) (Canetto, 2017). An Australian psychological autopsy case-control study found older adults are more likely to seek professional help, receive treatment and express their hopelessness and suicidality before death (De Leo et al., 2013). A population-level ecological study observed geographic variability in suicide mortality analyses – older men who immigrated from North-West Europe to Australia and were dwelling as tenants were more represented in suicide mortality (Law et al., 2015). An Australian study of young males (aged between 18 and 30 years) with psychiatric problems found this group to be disinclined to seek professional help following a serious suicide attempt (Cleary, 2016).

Some males at-risk for suicide seek help but many do not get it (resulting in negative outcomes) – many received services that are not well-tailored to men (Pirkis et al., 2016; Milner et al., 2017). An Irish qualitative study noted the rate of suicide is highest among middle-aged men in many high-income countries but there is a lack of knowledge with regards to psychological distress and support-seeking among this cohort (O’Donnell and Richardson, 2020). A plurality of constraining themes emerged for middle-age masculinities and mental health experiences: perception of increased expectation countered by diminishing capacities to achieve, isolation, shame in having to ask for help and failing in their independence (as barriers to seeking help) (O’Donnell and Richardson, 2020).

Epidemiological studies are providing a pathway to understanding suicide in men. A longitudinal cross-sectional study of psychosocial job stressors in Australian men found an overall high prevalence – there was an elevated presence of suicidal ideation in those with low job control, job insecurity, and unfair pay (Milner et al., 2017). Engagement with mental health services is increasing for men in Australia but many of those at-risk of depression and suicidality are prematurely dropping out without informing their clinician because of a lack of progress/tailored treatments and connection with the therapist (Seidler et al., 2021). Gender norms are being investigated in trials with boys and young men in Australia, Canada, New Zealand, and the United Kingdom to determine the effect of mental health literacy programs (e.g., focused on helping a friend in sporting club settings) and facilitate positive attitude development toward help-seeking for mental health (Rice et al., 2021). There are aims to broaden equity via socio-cultural adaptability of the programs (two school-based interventions and one online intervention).

The mechanisms for understanding men’s approach to mental health and suicidality was broadened by investigative insights from forensic screening, systemic review, sociological autopsy, qualitative, mixed method, and retrospective studies. A systematic review emphasized the opportunity to implement suicide prevention strategies during contact with primary health care – the average contact rate was 80% in the year prior to suicide and 44% in the final month before suicide (Stene-Larsen and Reneflot, 2017). However, screening and treatment of men’s mental ill-health is complex. Men are more likely to manifest atypical behaviors (e.g., abusive, aggressive, risk-taking, or antisocial behavior) (Streb et al., 2021) and apply violent or lethal methods of suicidal behavior as an extenuation of difficulties from not recognizing or reporting depressive symptoms (Rutz and Rihmer, 2021). A systematic review with meta-analyses of observational studies in the United States found that firearms were used in more than half of suicide attempts but there was inconclusive evidence that those with a diagnosis of a mental disorder were less likely of dying by suicide with a firearm (Zuriaga et al., 2021). Training for health care workers (Rutz and Rihmer, 2021) and screening tools for forensic psychiatrists (Streb et al., 2021) were suggestions for better detecting male depression and instilling broad awareness of how it atypically manifests.

A mixed methods study found older men from rural Australia had lower rates of diagnosis despite similar mental health rates (compared with women) (Fitzpatrick et al., 2021). The patterning of one’s life course was found to be related to suicidality with regards to inequality and marginality, perceptions of culturally normative autonomy, and unmet needs within a social care system. A qualitative study with rural men in Canada noted a paradox of higher rates of suicide and substance abuse that coincide with lower levels of stress and depression (Herron et al., 2020). Most men desire to talk about their mental health but were challenged by relationships and places in which to do so. Hegemonic masculinity was reported as being mostly resisted. The hindering issues to men obtaining mental health treatment in the rural Australian context include stigma, lack of emotional expression, non-disclosure of distress, and barriers to seeking and getting help (Kennedy et al., 2020). A retrospective study of bereaved men’s help-seeking before suicide found themes of complex relationships to seeking and getting help – there were those entrapped by secrecy and concealing the need for help, those with overwhelming illness that couldn’t be helped, as well as those whom services and systems provided ineffectual help (Oliffe et al., 2020).

## Current State of the Art – Digital Tools and Technology

Digital tools (e.g., video conferences, social networks, virtual rooms, telephone calls, emails, audio conferences, online intervention platforms, smartphone/tablet apps, online forums, and chats), as well as predictive, immersive and wearable technologies are emerging as promising as an adjunct to mental health care and suicide prevention but also hindered by challenges in their adoption and sustainment (American Psychological Association [APA], 2021) especially for young people (Wies et al., 2021). Previously, digital suicide prevention was noted as involving the use of machine learning, smartphone apps, and wearable sensor driven systems (Vahabzadeh et al., 2016). But it is unclear how feasible and scalable digital tools and technologies may be effectively used for suicide prevention (with suitable specifications for men).

### Data-Driven Identification and Intervention Approaches

Machine learning for suicide prediction emerged in a suicide risk algorithm study with US Army soldiers – 52.9% of posthospitalization suicides occurred after the 5% of hospitalizations with highest predicted suicide risk (Kessler et al., 2015). A pilot study applied an algorithm among patients with a mood disorder and identified suicide risk factors at the individual level by incorporating demographic and clinical variables (Passos et al., 2016). However, caution arose for recommending machine learning for suicide prediction (Chan et al., 2016; Mulder et al., 2016; Vahabzadeh et al., 2016). The clinical use of machine learning was noted as limited by an ongoing lack of information on model building and uncertain accuracy (Graham et al., 2019; Moon et al., 2019; Shatte et al., 2019) which contribute to potential ethical and legal issues (Balcombe and De Leo, 2021a; Schueller, 2021; Wies et al., 2021).

A natural language processing algorithm reliably identified risk for suicide and assisted telehealth clinicians to promptly respond with crisis resources (Bantilan et al., 2020). A lack of empirical evidence (Thieme et al., 2020) and a lack of efficacy evaluation in experimental and feasibility studies has challenged effective implementation of algorithms (Fonseka et al., 2019; Haines-Delmont et al., 2020; Roy et al., 2020). A commentary outlined that machine learning has outstanding potential, but it is constrained by insufficient data resulting in a lack of generalizability and sufficient comprehensiveness with regards to relevant variables (Lennon, 2020). The human-centered artificial intelligence (HAI) framework promotes ethically aligned design, human factors design and technology enhancement in a refocus on user-centered design processes (Xu, 2019) that have hindered human-computer interaction (Thieme et al., 2020).

Various population studies with machine learning algorithms have yet to be externally validated. A Swedish population study investigated national registry data of inpatient and outpatient visits with models trained for suicide attempt/death within 90 and 30 days following a visit (results were 3.5 and 1.7%, respectively) (Chen et al., 2020). A Danish population study found that machine learning may advance prediction of suicide (especially men with depression prescribed non-opioid analgesics, antipyretics, hypnotics, sedatives, and diagnosed with a poisoning) (Jiang et al., 2021). Two recent studies proposed advanced suicide prediction models (with machine learning methods that identify subgroups) in a significant prospective cohort study of the United States population (García de la Garza et al., 2021; Machado et al., 2021). A main difference between the studies is hypothesis-driven methods of the former study and data-driven approach of the latter study. This highlights a challenge of integrating machine learning algorithms with standard clinical care – the convergence of hypothesis-driven and data-driven methodologies is hindered by different evaluation approaches (i.e., traditional and modern) (Balcombe and De Leo, 2021a). For example, the agility of machine learning means it is redundant for a long series of RCTs (Shatte et al., 2019). A call was made for simulation-based approaches to assist end-users in expediting their evaluations (Guo et al., 2020).

Trials of technology-enabled models of care for Australian youth are seeking to demonstrate efficient and comprehensive coordination and delivery of mental health services (e.g., improving pathways to care and response to suicidal behaviors in a local system dynamics model) (Iorfino et al., 2021). An agent-based model and discrete-event model were applied for an individual-centric approach to seek appropriate services, follow a pathway and receive treatment. A simulation provides a comparison of a “business as usual” approach and a technology-enabled care approach to assist in the evaluation of whether the “right” level of effectiveness is being delivered. Algorithms provide a fast and consistent response to risk by reducing “business as usual bottlenecks” via standardized digital pathways that determine appropriate care for those with high needs (Iorfino et al., 2021). The treatment frequency and reviews are determined by needs – suitable intervention targets are set by the model then it reviews targets over time to inform better care decisions. This online multi-dimensional assessment and triage is a personalized model of care that assists clinicians early in the intake to accelerate an automated process (and in effect reducing duplication).

Machine learning (including deep learning) has been proposed as promising for easing the understanding of mental health (between the patient, mental health care practitioner and the machine) (Barredo Arrieta et al., 2020). A proof-of-concept prototype development for an early intervention study suggested application of explainable artificial intelligence (XAI) by combining common sense knowledge with semantic reasoning and causality models (Ammar and Shaban-Nejad, 2020). However, the temporality of suicide means there is a risk of machine learning becoming outdated because of a lack of specificity for data points from registries, electronic health records or other databases (Lennon, 2020). There is emerging evidence using radiography or online data methods. A cross-sectional assessment of structural Magnetic Resonance Imaging (MRI) data with a support vector machine learning model for a sample of adolescents/young adults diagnosed with major depression disorder found a high-level of accuracy for predicting those with suicide ideation and/or attempts (Hong et al., 2021). A transformer-based deep learning model sought suicide risk identification from social media sources – it was proposed as effective for classifying suicide notes (i.e., contextual and long-term dependency information determined from different datasets) (Zhang et al., 2021). Machine learning helps analyze big data easier by automating processes – this helps support a better predictive potential of an individual’s suicide risk but there is yet to be accurate prediction of specific risks across populations.

### Internet and Related Technologies

The most common digital tools used for self-help are accessed online via digital platforms and apps. Guided approaches are delivered in web chat and peer support forums as well as by phone (e.g., calls or texts) to provide crisis support, psychotherapy, counseling, psychological treatments, as well as support for recovery. Health promotion, education, and mental ill-health/suicide prevention are available in self-help or guided approaches.

The evidence synthesis on self-help suggests a suitability for suicidal men in quickly helping themselves and getting linked to unobtrusive services and support when needed. A RCT found that web-based self-help can be effective in reducing suicidal ideation and serves well as an adjunct to regular care (57% of participants were receiving additional help) – embedding the intervention in an integrated online suicide prevention platform helps to provide human support by email or a quick referral to more intensive care if required (Van Spijker et al., 2014). Three RCTs with a brief, mobile intervention (a game-like app) found promising reductions in suicidality with note of a potential to have a large-scale impact (Franklin et al., 2016). A systematic review concluded self-guided digital interventions for suicide prevention are effective (at least in the short-term), should be promoted and integrated into health systems in countries where it has been tested (Torok et al., 2020). But there needs to be population studies, alleviation of safety concerns as well as further studies for non-CBT approaches being possibly more effective (Torok et al., 2020).

Head to Health is an Australian digital platform bringing together more than 700 trusted mental health services (i.e., apps, online programs, online forums, and phone services, as well as a range of digital information resources). It is noted as effective for people with or at risk of mild to moderate mental health difficulties. There are 41 resources for suicide prevention (two apps, two forums, six phone call links, five web chat links, two email links, one SMS link, one video call link, 19 websites, and three information resources on suicidal thoughts and finding support, crisis links as well as information for parents). There are no known studies that have explored the demographics of Head to Health (or other integrative digital platform) users or the efficacy and impact of its use. A RCT evaluated the usability of the Thought Spot digital platform – a high attrition rate was noted with only 20% of mental health services or resources viewed by end users (Shi et al., 2021). Connection and engagement in a therapeutic alliance is important in using digital platforms (Arshad et al., 2019). Although the overall effect size of suicide prevention digital interventions is minor, there is an opportunity to explore the population impact if promoted though internet and digital distribution platforms (e.g., it is possible through app stores) (Torok et al., 2020). A qualitative study focused on virtual coaches for CBT noted engagement difficulties hindering the effectiveness of using digital platforms (Venning et al., 2021). Guided approaches for suicide prevention are useful if carefully administered and delivered but require more efficacy. Telehealth use in primary care (i.e., a suicide prevention hotline) appeared to have temporal efficacy (Rhee et al., 2005). A clinical trial found telepractice to be as feasible as traditional in-office clinical care delivery if there is accordance with safety plans, training, and published guidelines (Luxton et al., 2014). Telehealth was deemed as useful in continued psychiatric care during the COVID-19 pandemic especially for postvention (Rothman and Sher, 2021). A systematic review and random-effects meta-analyses of brief contact interventions (letter, postcard, telephone, green card or crisis card) for reducing self-harm, suicide attempt, and suicide recommended well-designed trials in clinical populations (Milner et al., 2015). A RCT involving caring emails intended on preventing suicide behaviors among United States service members and veterans (Luxton et al., 2019) also did not draw firm conclusions on efficacy but no adverse effects were associated with the intervention.

Qualitative research has been applied in mixed self-help and guided approaches for a mental health promotion context highlighting the potential of a positive impact through novel, media-based public health interventions. A trial involved an online survey that measured the impact of the Man Up documentary, an Australian media-based public health intervention that explored the relationship between stoic masculinity, mental health and suicide - key messages included being prepared to help others and being more emotionally expressive (King et al., 2018). Testing of the promotional materials and website for the Man Up program found active and empowering language to be potentially useful for informing men’s mental health promotion interventions (Schlichthorst et al., 2019).

### Digital Interventions and Technologies

Digital interventions for suicide prevention include automated CBT, digital speech analysis, facial emotion analysis, smartphone/mobile apps, wearable sensors and cloud-based technology (Vahabzadeh et al., 2016; Rege, 2020) as well as a possibly more effective combination of dialectic behavior therapy, CBT for insomnia, and therapeutic evaluative conditioning (Torok et al., 2020). Mobile/smartphone apps have a large share of the technology market but there are issues with the privacy of personal data, and the need for clinical guidance and education about the lack of evidence (Torous et al., 2018).

Although technologies are rapidly advancing, empirical evidence for digital interventions is lagging. A rapid meta-review found digital interventions are well-disposed to mitigate mental ill-health at the population level with good evidence on usability, safety, acceptance, satisfaction, and effectiveness (Rauschenberg et al., 2021). But there are limited guidelines on interventions for patients who identify as high risk of suicide on digital platforms (Bai et al., 2020; Bailey et al., 2020). A systematic review found social media platforms, e-learning content, online resources and mobile apps were developed to deliver digital interventions during COVID-19 but were limited by a low level of empirical evaluation, challenges in adoption as well as a need for greater heterogeneity (Drissi et al., 2021). A systematic review of digital interventions for indigenous people found an overall acceptability of an array of digital technologies with promising measurable outcomes to help address high rates of suicide, depression, and substance abuse (Li and Brar, 2021). A comprehensive review of digital interventions for suicide prevention found a great potential to advance the response to suicide risk but standalone digital interventions are likely insufficient because apps or media present a single suicide prevention strategy (lacking integration with others on a platform) (Braciszewski, 2021).

A narrative review of multiple RCTs and a large meta-analysis of the effectiveness of digital psychotherapy found good evidence for depression and anxiety disorders of mild-to-moderate severity (Weightman, 2020). But the evidence of effectiveness for other digital interventions is not as clear. Togetherall (previously Big White Wall) is a global online mental health support community (peer-to-peer approach moderated by clinical professionals). It was the subject of a fully automated RCT which sought to test the clinical effectiveness of the online peer-support program versus web-based information for self-management of anxiety and depression (Morriss et al., 2021). However, there was insufficient recruitment and retention of participants – a personal approach to participant engagement was recommended. Controlled studies are required to increase the effectiveness of CBT in reducing suicidal ideation (Büscher et al., 2020) but it is the most used digital intervention (Van Doorn et al., 2021).

A systematic review found digital interventions and technologies used in counseling are as effective as face-to-face sessions (De Bitencourt Machado et al., 2016). Quality assurance for digital interventions was proposed as applicable to the existing arrangements with governing agencies or professional associations (Dores et al., 2018). An online survey of the use of digital technologies in psychological counseling before and during a COVID-19 lockdown noted a slight rise in the use of video conferences, social networks and virtual rooms but slightly less use of telephone calls, emails, audio conferences, online intervention platforms, smartphone/tablet apps, online forums, and chats (Dores et al., 2020).

A case series on young adults with a psychotic illness found digital phenotyping via tracking on a smartphone app is a feasible way to monitor and detect patient status as opposed to an intervention (Wisniewski et al., 2019). Limited by individualized evidence, population studies were recommended to increase the potential for digital phenotyping to monitor in real time for safety (e.g., risk of suicide) (Wisniewski et al., 2019). Digital phenotyping of passive data from smartphone and wearables in addition to questionnaires could potentially measure the suicidal ideation process through ecological momentary assessment (Braciszewski, 2021). Rather than focus on the empirical value of digital phenotyping, an analysis found a good potential on practical, efficient, convenient, and non-intrusive information acquisition about holistic health as well as a potential to provide valuable insights related to the various factors that constitute a diagnosis, state or condition (Coghlan and D’Alfonso, 2021).

### Immersive Technologies

Virtual reality (VR) was applied in experiments to test ideas about the causes of suicide – males were more likely to have VR suicide completion, but the translational approach was noted as an approximation of actual suicide which largely limits its ecological validity and makes it only tentatively useful (rather than replicable) for future studies to gain knowledge about suicide (Franklin et al., 2019). Subsequent research has mainly focused on the mental health implications of youth using VR and video games. A systematic review of a small number of available digital interventions for common disorders and phobias in children and young people via video games and VR found evidence of efficacy but noted the need for co-design, development, and evaluation before they are used in treatment (Halldorsson et al., 2021). A longitudinal approach to development and design, and greater heterogeneity are required for studies evaluating immersive digital interventions for children (Davies and Bergin, 2021). A systematic review of the use of commercial off-the-shelf video games (i.e., exergames, casual video games, action games, action-adventure games, and augmented reality games used in consoles, PCs, smartphones, mobile consoles, and VR systems) suggested there is effectiveness in reducing stress and anxiety (Pallavicini et al., 2021).

## Highlight of Future Directions

Future studies for suicide prevention in men should investigate the efficacy of self-help approaches as a core issue and explore the potential of branching out the ecosystem with adjunct digital tools and technology including the validation of machine learning algorithms in different populations, the development and use of high quality and high impact integrated digital platforms as well as optimization of technology-enabled care coordination (e.g., automated personalized model of care to link patients efficiently and effectively with appropriate mental health care services).

## Key Concepts

Suicide continues to contribute to a globally significant socio-economic burden. Suicidality is exemplified in men of all ages, notably during middle-age but older men have also represented the highest risk. Although mental ill-health is a significant risk factor, its accurate prevalence is unclear as many men are not intervened because of their self-reliance and stoicism as well as ineffective mental health care treatments (complicated by atypical behaviors and manifestations of depression). There have been promising signs of improvements with men engaging in mental health care services and promotion interventions but accurately predicting and intervening their suicidal behavior has complexities and challenges. The potential of applying machine learning to population datasets to predict suicidal behavior remains unknown. Automation has led to advances of efficacy at the individual level but engagement with subjective experiences remains crucial for mental health care practitioners. Digital tools and online resources are available for self-help or guided approaches via digital platforms and apps, web chat, peer support forums, email and/or phone as well as digital interventions (e.g., mainly CBT but also digital phenotyping as well as VR and video games).

The evidence synthesis found higher levels of efficacy and evaluation are required before recommending digital interventions for distress and suicidality. But there is a good potential for self-guided digital interventions to serve as an adjunct especially with men (who may often find it difficult to get satisfactory help and can be complex and difficult to serve). It is unclear what to prescribe for digital suicide prevention because of a lack of strategy integration for apps or media – there is potential for better human-computer interaction (e.g., promoting suicide prevention apps on internet and digital distribution platforms). Well-designed and developed multi-modal digital platforms may help to advance evaluation of online resources for suicide prevention, stimulate evidence for long-term suicidal behavior prediction and optimal interventions as well as serve as a hub for self-help, digital interventions and technology-enabled care coordination.

## Author Contributions

LB contributed to the planning, conduct, and reporting. DD contributed to its review. LB and DD contributed to the final article and approved the submitted version of the manuscript.

## Conflict of Interest

The authors declare that the research was conducted in the absence of any commercial or financial relationships that could be construed as a potential conflict of interest.

## Publisher’s Note

All claims expressed in this article are solely those of the authors and do not necessarily represent those of their affiliated organizations, or those of the publisher, the editors and the reviewers. Any product that may be evaluated in this article, or claim that may be made by its manufacturer, is not guaranteed or endorsed by the publisher.
